# A quality improvement project to reduce magnetic resonance imaging sedation in children

**DOI:** 10.1007/s00247-025-06293-4

**Published:** 2025-08-06

**Authors:** Juan Boriosi, Christina Bryndzia, Michael Lasarev, Justin Brucker, Susan Rebsamen, Teresa Chapman, Brianna Peterson, Megan Peters

**Affiliations:** 1 University of Wisconsin–Madison, H6/562 CSC, 600 Highland Ave, Madison, WI 53792 United States; 2 UW Health Kids, Madison, United States

**Keywords:** Anesthesia, Child, Hypnotics and sedatives, Magnetic resonance imaging

## Abstract

**Background:**

Our institution decided to implement an awake MRI scanning quality improvement project using audiovisual distraction (AVD) technology.

**Objective:**

To reduce the utilization of minimal/moderate sedation by at least 20% in children 4 to 18 years, while maintaining comparable diagnostic quality and adhering to allotted exam times, through the implementation of an awake MRI program.

**Materials and methods:**

This project was conducted at a pediatric sedation clinic between October 2021 and January 2024. We included patients 4 to 18 years of age, scheduled for an MRI at the pediatric hospital, and eligible for either minimal/moderate sedation or AVD. The outcome measure was the percentage of patients referred to our sedation clinic who completed an MRI with AVD and without sedation, analyzed on a statistical process control (SPC) P-Chart. Process measures were the number of children eligible for AVD per month, analyzed on an SPC C-Chart. Balance measures were the number of studies that exceeded allotted exam time or were non-diagnostic.

**Results:**

Of 734 MRI referrals aged 4 to 18 years, 320 patients met inclusion criteria. Two hundred twenty-eight (71.3%) received minimal/moderate sedation (mean age [SD] 9.7 years [± 3.0]) and 92 (28.8%) underwent AVD (mean age 10.0 years [± 2.7]). The average monthly decrease in minimal/moderate sedation use was 28.8 percentage points. The average number of children considered eligible for AVD increased by special cause variation from 3.8 to 7 patients per month. All 92 MRI referrals triaged to AVD completed their MRI successfully without sedation; all studies were diagnostic, and 96% of studies were within the allotted exam time.

**Conclusion:**

We implemented an awake MRI program with AVD that decreased monthly sedation needs by 28.8 percentage points while maintaining a high rate of diagnostic studies and no MRI delays.

## Introduction

Children under age 8, with developmental delay or autism spectrum disorder, may require sedation or general anesthesia (GA) to remain motionless and tolerate the magnetic resonance imaging (MRI) scanning process, depending on scan type. However, sedation and GA have risks of respiratory depression and prolonged hospital visit time [1]. Recently, there has been an increasing concern regarding sedation/anesthesia-related neurotoxicity in the pediatric population [2].

There are several strategies to minimize sedation in children undergoing MRI studies [3]. Optimization of the MRI environment with age-appropriate themes may decrease the need for sedation and GA in children 4–6 years old [4]. Mandatory evaluation by a certified child life specialist (CCLS) before MRI may reduce the need for GA by 14.8% in children 5 to 18 years [5]. The implementation of a mock MRI scanner to familiarize the child with the machine and noise resulted in a 16.1% decrease in the use of GA for children ages 3 to 8 years [6]. Feeding-and-bundling is a non-sedation option highly effective in neonates, with a success rate for brain MRI ranging from 90 to 95% [7]. Noise-reduction strategies include the use of diminished gradient slew rate and slow ramps for k-space readout in combination with an ultrashort echo time, insulation of the inner bore of the magnet, and inserting earplugs and covering the ears with noise attenuators [8]. Additional strategies include using imaging techniques that can decrease scanning time, like parallel imaging, simultaneous multisection imaging, radial k-space acquisition, compressed sensing MRI reconstruction, and automated protocol selection software [9]. Most recently, artificial intelligence (AI)-based algorithms, particularly deep learning models, are being used to reduce scan times by reconstructing high-quality images from significantly fewer data points [10]. MRI-compatible audiovisual distraction (AVD) technology was introduced in clinical practice at the beginning of the century and has been reported to decrease the use of sedation in children by 15–35% [11–13].

Our institution had previously lacked MRI-safe AVD technology and decided to implement an awake MRI scanning quality improvement project using new open-bore MRI AVD technology. In the year 2021, before the availability of MRI AVD, our sedation clinic received 339 MRI referrals in patients aged 4 to 18 years. Of those referrals, 92% (*n* = 311) were sedated; 45% (*n* = 140) received minimal or moderate sedation, and 55% (*n* = 171) received deep sedation. Only 6% (*n* = 20) of MRI referrals completed their study successfully without sedation. Given this data, a quality improvement project was implemented whose primary objective was to reduce the utilization of minimal and moderate sedation by at least 20% in children 4 to 18 years old undergoing pediatric MRI at our sedation clinic, while maintaining comparable diagnostic quality and adhering to allotted exam times through the implementation of an awake MRI program using AVD technology.

## Material and methods

### Context

This quality improvement project was conducted between October 2021 and January 2024 at an inpatient and outpatient pediatric sedation clinic in an urban academic quaternary care children’s hospital, wherein the children’s services occupy a separate but attached pediatric hospital, and MRI scanners and technologists also serve adult patients in the neighboring university hospital. Our clinic provides minimal, moderate, and deep (i.e., propofol) sedation defined as a medically induced alteration in consciousness with the intent to maintain a natural airway. Patients deemed at risk of airway instrumentation with the administration of sedatives are usually referred to GA. The project followed Plan-Do-Study-Act (PDSA) cycles: an iterative problem-solving model used for improving health care processes. The cycle begins with making predictions about the outcome (Plan), conducting the plan and collecting data (Do), comparing the predictions to the data collected after specific interventions (Study), and acting based on the new knowledge, validating whether the hypothesis for improvement is correct (Act) [14]. The sedation (physicians, schedulers, certified child life specialist, and nurse manager) and radiology teams (physicians, radiology manager, and MRI technologists) were involved in these cycles. Patients referred to the pediatric sedation clinic for a sedated MRI were screened for the study. Patients with visual impairment, severe developmental delay, or severe autism spectrum disorder that would preclude understanding of the AVD technology or tolerance of the confined MRI space were excluded from the study. The inclusion criteria for this study changed, broadening successively for diagnosis, age, and scanning time, with each PDSA cycle undertaken by the stakeholder team. This project followed the Revised Standards for Quality Improvement Reporting Excellence (SQUIRE 2.0) and was exempt by the local institutional review board [15].

### Planning the intervention

**Fig. 1:**
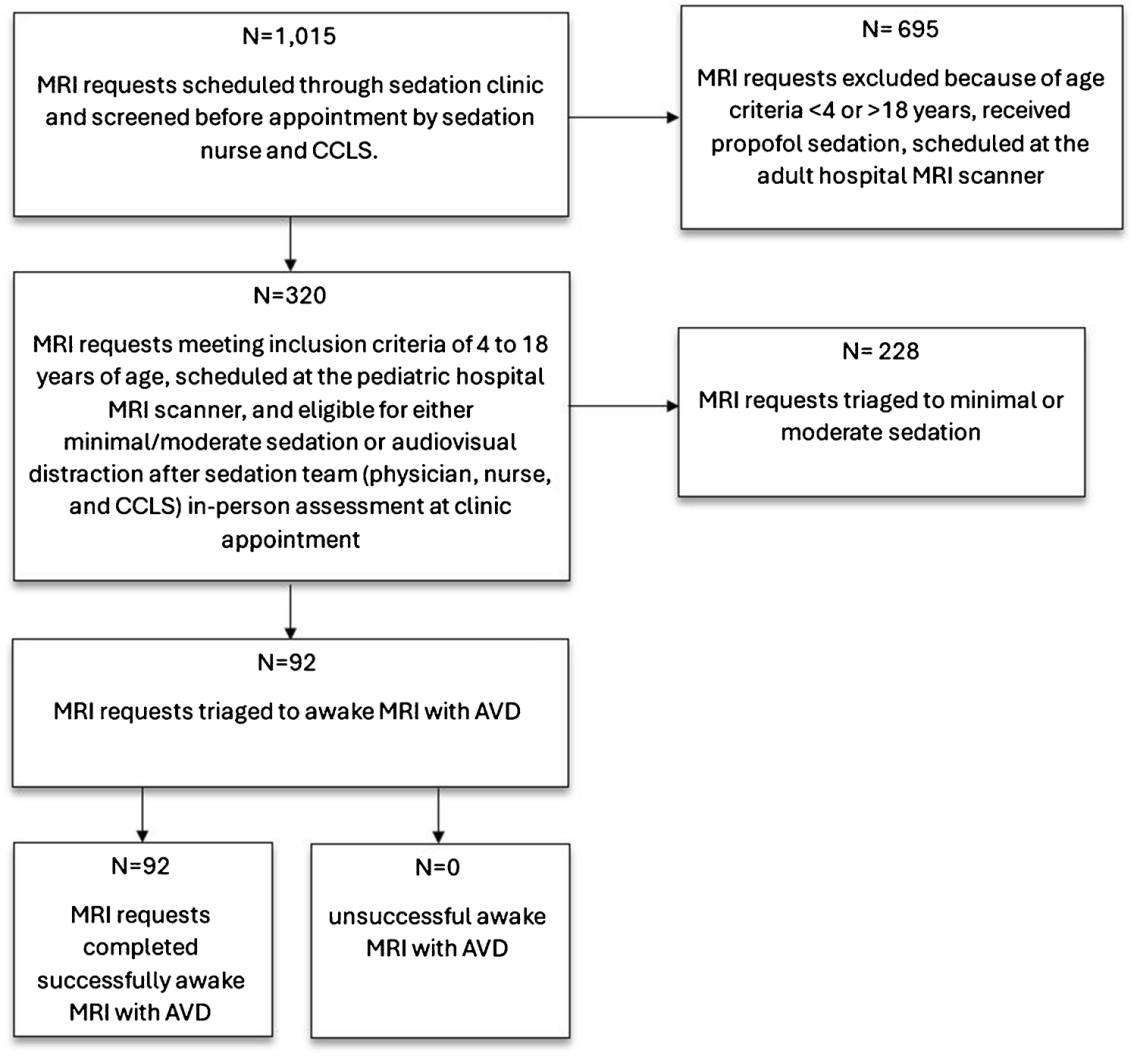
Awake MRI workflow

In October 2021, a multidisciplinary stakeholder team, including the sedation clinic director and radiology personnel, convened to develop a new workflow for an awake MRI program (Fig. 1 and Table 1).

**Table 1:** Detailed description of Plan-Do-Study-Act cycles undertaken by stakeholder team

	Dates	Description of interventions
Planning the intervention	10/2021–12/2021	Convened stakeholder group Performed retrospective chart review and collected pre-implementation data Established outcome, process, and balance measures
Targeting the intervention PDSA cycle #1	1/2022–4/2022	Installed MRI in-bore video system. PDC Inc. Hartland, WI Developed workflow for awake MRI program Established “pilot” awake MRI program criteria: 7 years and older, with diagnosis of central nervous system tumor, needing follow-up MRI scans, head-first MRI position only, and less than 60 min scan duration
Broadening the scope for diagnosis PDSA cycle #2	5/2022–8/2022	Broadened inclusion criteria to any diagnosis except patients with visual impairment, severe developmental delay, or severe autism spectrum disorder who were still excluded from the study
Broadening the scope for age PDSA cycle #3	9/2022–3/2023	Broadened inclusion criteria to age 4 years and older
Increasing likelihood of a diagnostic study PDSA cycle #4	4/2023–8/2023	Implemented neuroradiology motion sensitivity score Eliminated any restrictions for MRI duration (i.e., all head-first MRIs eligible for awake MRI scanning)
Reinforcing the use of AVD at the bedside PDSA cycle #5	9/2023–1/2024	Installed new technology MRI view. MRI Audio. Carlsbad, CA Re-educated stakeholder group about new technology that allowed scanning in any body position Culture change to reinforce the value of awake MRI among stakeholders

Various MRI AVD technologies were evaluated in terms of cost and convenience, and a decision was made to trial an open-bore technology (MRI in-bore video system, PDC Inc. Hartland, WI) for the MRI scanner located in the pediatric hospital. This technology consists of a video projector located outside the MRI bore that projects a movie onto the upper inner surface of the bore. This technology was suitable only for head-first positioning in the MRI scanner, the most common study type performed at the imaging center located in the pediatric hospital. The AVD technology was installed and available for clinical use at the children’s hospital MRI scanner on January 27, 2022.

### Targeting the intervention (PDSA cycle 1)

The patients receiving minimal and moderate sedation (i.e., non-propofol sedation) were targeted for the awake MRI program because patients receiving propofol (deep sedation) usually have anxiety or are developmentally unable to cooperate with the use of the AVD technology. The team created an “awake MRI” workflow to outline the criteria to use the AVD technology in patient care (Fig. 1). Patients referred to the sedation clinic for a sedated MRI were identified as eligible candidates by the sedation staff for our awake MRI program before arrival at the clinic, by review of clinical information and direct communication with the family by phone. At the clinic visit, on the day of the sedation/MRI exam, a CCLS, a nurse, and a sedation provider performed an in-person assessment on all eligible patients to trial awake versus sedated MRI (Fig. 1 and Table 2). Patients who failed an awake MRI were immediately sedated at the same-day appointment.

**Table 2:** Certified child life specialist (CCLS) assessment for pediatric awake versus sedated MRI

Pre-appointed review of child’s medical record	In-person assessment during MRI appointment	In-person preparation and education during MRI appointment
Focusing on age, developmental level, any sensory disorders, autism, or behavioral disorders. Review of past radiology experiences (MRI, CT scan)	Focusing on child’s temperament, demeanor, verbal and body language, and understanding of their MRI experience	• Assessment of the child’s behavior, body language, and anxiety level with the procedure after education and preparation • The CCLS, nurse, and sedation provider (physician or pediatric nurse practitioner) decide for awake versus sedated MRI based on the assessment provided by the CCLS

Criteria to use AVD were age 7 years and older, diagnosis of central nervous system tumor, needing follow-up brain MRI scans, head-first MRI position only, less than 60 min scan duration, and MRI scheduled at the children’s hospital. This was primarily to maximize the diagnostic quality of MRI imaging and to minimize the likelihood of a patient needing to convert to a sedated scan, thus prolonging the allotted exam time, defined as scan time plus 15 min to account for patient and room set-up.

### Broadening the scope for diagnosis (PDSA cycle 2)

The sedation team (sedation provider, nurse, and CCLS) noted that their in-person assessments of patients were yielding more patients who would likely benefit from AVD than the initial narrow criteria allowed. Therefore, the criteria to use AVD were broadened to cases for non-CNS indications. However, patients with visual impairment, severe developmental delay, or severe autism spectrum disorder that would prevent understanding of AVD technology and tolerance of confined MRI space were still excluded from AVD use. This change in diagnostic criteria allowed the use of AVD for any type of head-first MRI with a scan duration of less than 60 min, scheduled at the children’s hospital.

### Broadening the scope for age (PDSA cycle 3)

This PDSA cycle reduced the restrictions for age to any patient 4 years and older who was found to be cooperative, calm, and likely to remain still without sedation on the day of their MRI based on CCLS, nurse, and physician in-person assessment.

### Increasing likelihood of a diagnostic study (PDSA cycle 4)

This PDSA cycle removed restrictions on scan duration, allowing examinations of any length. In addition, alongside the ongoing work to reduce sedation for MRI scans in the summer of 2022, the pediatric neuroradiology team developed an array of accelerated MRI protocols that reduced both the time to acquire each sequence and the total time spent in the MRI scanner, thus optimizing the chance for success. Several complex protocols, however, remained, involving detailed imaging that required longer scan and sequence times, particularly for patients undergoing evaluation for seizure focus resection. Because of the importance of these images for surgical planning, and because individual sequences in these protocols could stretch beyond 10 min in duration, the pediatric neuroradiology team was very interested in minimizing the number of patients with motion artifact on these exquisitely motion-sensitive studies. In the spring of 2023, they developed and distributed a motion sensitivity score (Table 3) awarding each protocol a “difficulty” score based on scan parameters by which the sedation team could triangulate the motion sensitivity score of the MRI protocol alongside a bedside assessment of the patient’s temperament and cooperability on the day of their appointment. After these scoring systems were integrated into the sedation workflow, they were integrated by the pediatric radiology department supervising MRI protocols for pediatric patients, unifying the use of the motion sensitivity score, which was also used to help decide on patients that were appropriate for AVD. In addition, an average of 30 min was added to the allotted exam time at the time of scheduling to allow sedation of patients who failed an awake MRI due to motion artifact.

**Table 3:** Pediatric MRI exams, allotted exam times, and assigned motion sensitivity scores. Scanning times include a 15-min allowance for room set-up prior to scanning

Pediatric MRI exam type	Allotted exam time (minutes)	Motion sensitivity score*
Quick exams of any body part (limited MR exams utilizing fast T2-weighted sequences, fast T1-weighted sequences, and diffusion-weighted imaging)	15	0
Fast brain exams (rely on sequences with accelerated acquisitions)	30	0
Fast/feed/swaddle neonatal brain exams	30–45	0
Accelerated total spine exams, routine single-level spine exams	30–60	1
All non-CNS body and MSK single-part exams	30	2
Brain MRI either without contrast or with IV contrast	45	2
Total spine MRI without contrast	90	2
MR angiography without contrast	30–45	2
MR angiography with contrast	60	3
Brain MRI with advanced sequences (e.g., DTI, phase-contrast craniocervical CSF flow)	60	3
Brain + spine MRI without contrast	60	3
Spine MRI, 2 or more levels, without and with contrast	90	3
Head and neck protocols	60–75	3
Brain + spine MRI without and with contrast	90	4
Brain MRI with critical high-resolution sequences (e.g., high-resolution epilepsy protocol)	75	4
Head and neck protocols with vascular or high-resolution nerve imaging	75–90	4

^*^Motion sensitivity score meaning:

0 = limited MRI exams, no sedation required, bypasses sedation workflow

1 = accelerated or short exams less than 60 min, good chance for success without sedation

2 = routine exams that approach or exceed 60 min, and longest sequences within the exam are less than 4 min each, moderate chance for success without sedation

3 = total exam time exceeds 60 min and/or combined protocols and/or longer sequences are multiple, poor chance for success without sedation

4 = total exam time exceeds 60 min, multiple critical high-resolution sequences that exceed 4 min each, virtually no chance of success without sedation

*CNS*, central nervous system; *CSF*, cerebrospinal fluid; *DTI*, diffusion tensor imaging; *DWI*, diffusion-weighted imaging; *MSK*, musculoskeletal

### Reinforcing the use of AVD at the bedside (PDSA cycle 5)

Although the study team was able to remove barriers that limited patients’ access to AVD during previous PDSA cycles, it was noted in the spring of 2023 that the percentage of patients placed in the AVD pathway had fallen. Sedation team leaders and pediatric radiologists convened and shared run chart data with the CCLS and sedation nurses, directing their attention to very high success rates of patients distracted with AVD alongside the unexpectedly decreased rates of use of this tool in recent months. A culture change was encouraged to reinforce the value of awake AVD MRI among stakeholders.

In September 2023, the MRI in-bore video system, PDC Inc. Hartland, WI, was replaced with a new open-bore technology (MRI view, MRI Audio, Carlsbad, CA). This technology consisted of a self-standing mirror and projector and allowed the patient to watch a movie during head-first and feetfirst body position MRIs, which expanded the availability of AVD during MRI scanning.

### Study of the improvement

The quality improvement team continually reviewed prospectively collected data of all patients 4 years and older who were referred to the sedation clinic for MRI scanning at the pediatric hospital. We reviewed patient demographics, diagnosis, type of MRI, location, sedative use, AVD technology use, MRI duration compared to allotted exam time, defined as scan time plus 15 min to account for patient and room set-up, and study outcome (i.e., diagnostic or non-diagnostic). The data were reviewed monthly by the multidisciplinary team, and any changes were discussed and approved by all stakeholders.

### Measures

The primary outcome measure was the percentage of referrals who completed an awake MRI with AVD and without the need for sedatives, analyzed on a statistical process control (SPC) P-Chart, a type of control chart where each item can be categorized as either “pass” or “fail”—typically when subgroup sizes vary across data collection points [14]. This percentage was calculated using “all eligible patients for AVD” (i.e., minimal and moderate sedations for MRI and awake patients undergoing MRIs with AVD without sedatives) as the denominator and awake patients undergoing MRIs with AVD without sedatives as the numerator. Patients who received propofol sedation for MRI were excluded from the calculation of the primary outcome because they usually had developmental or behavioral statuses that would prevent their success with awake MRI. The process measure to track compliance with our awake MRI program workflow was the number of patients deemed eligible for AVD per month by inclusion criteria, analyzed on an SPC C-Chart, a type of control chart used to count items in a process at regular intervals [14]. A diagnostic quality awake MRI was defined as a study that did not require sedation to achieve diagnostic images. This was determined by the interpreting radiologist at the time of imaging, and as part of this quality improvement project, this was recorded in each dictation report’s technique section for all pediatric imaging studies during the study period, regardless of whether the exam was done with sedation or while awake. The balance measures, the metrics used to assess the unintended consequences of changes with the implementation of our awake MRI program, were the number of MRIs that were not of diagnostic quality or which required longer than the allotted exam time (Table 3). We chose this balancing measure because unanticipated MRI delays that prolong allotted exam time can significantly impact MRI workflow. Exam beginning and ending times were acquired from routine records created by the MRI technologists in the electronic health record.

Caregiver and patient satisfaction data were collected in a subset of patients following the conclusion of an awake MRI appointment. Patients were verbally asked, in language appropriate for their age, to rate whether their experience was “easy” (i.e., high satisfaction), “medium” (i.e., moderate satisfaction), or “hard” (i.e., low satisfaction). Caregivers were asked to rate their level of satisfaction on a Likert scale, with “1” representing “very dissatisfied” and “5” representing “very satisfied.”

### Analysis

Outcome and process measures were assessed with statistical process control chart methodology. Statistical process control charts were constructed with QI Charts (version 2.0.23; Performance Improvement Products, Austin, TX) and Microsoft Excel (version 16.73; Microsoft Corporation, Redmond, WA). Processes that are “in control” have stable and predictable behavior over time, and they are indicated by a stable centerline, which depicts the mean of the data over time. When a system or a process changes, either by project interventions or by external forces, the process is said to be no longer “in control,” and the centerline shifts to accommodate a new mean. Special cause variations are causes of variations in a process that are not inherent to the process. Means were shifted when corresponding to interventions that signaled special cause variation. Any of the following rules indicated special cause variation in SPC charts: a single data point outside of the control limits, eight consecutive points above or below the mean line, or a trend of six consecutive points all moving in the same direction, and two out of three points falling in the outer third of a control limit [16]. Continuous characteristics of the MRI referrals were summarized in terms of the range, mean, and standard deviation, or the median and interquartile range (IQR); categorical factors were described using frequencies and percentages. Data were described separately according to group (awake or sedated during the MRI), and comparison of characteristics between groups was made using *t*-tests, rank-sum tests, or chi-square tests. A *P*-value of less than 0.05 was considered statistically significant.

## Results

From January 2022 to January 2024, there were a total of 1,015 patients scheduled for a sedated MRI through our clinic. Of those, 734 MRI referrals were for patients 4 to 18 years of age and met study age inclusion criteria. Eighty-three percent (*n* = 606) of patients had sedation; 13% (*n* = 92) of patients had AVD without the need for sedatives; 3% (*n* = 20) required neither sedation nor AVD for their MRI; and 2% (*n* = 16) of scheduled patients were cancelled (before sedative administration).

**Fig. 2:**
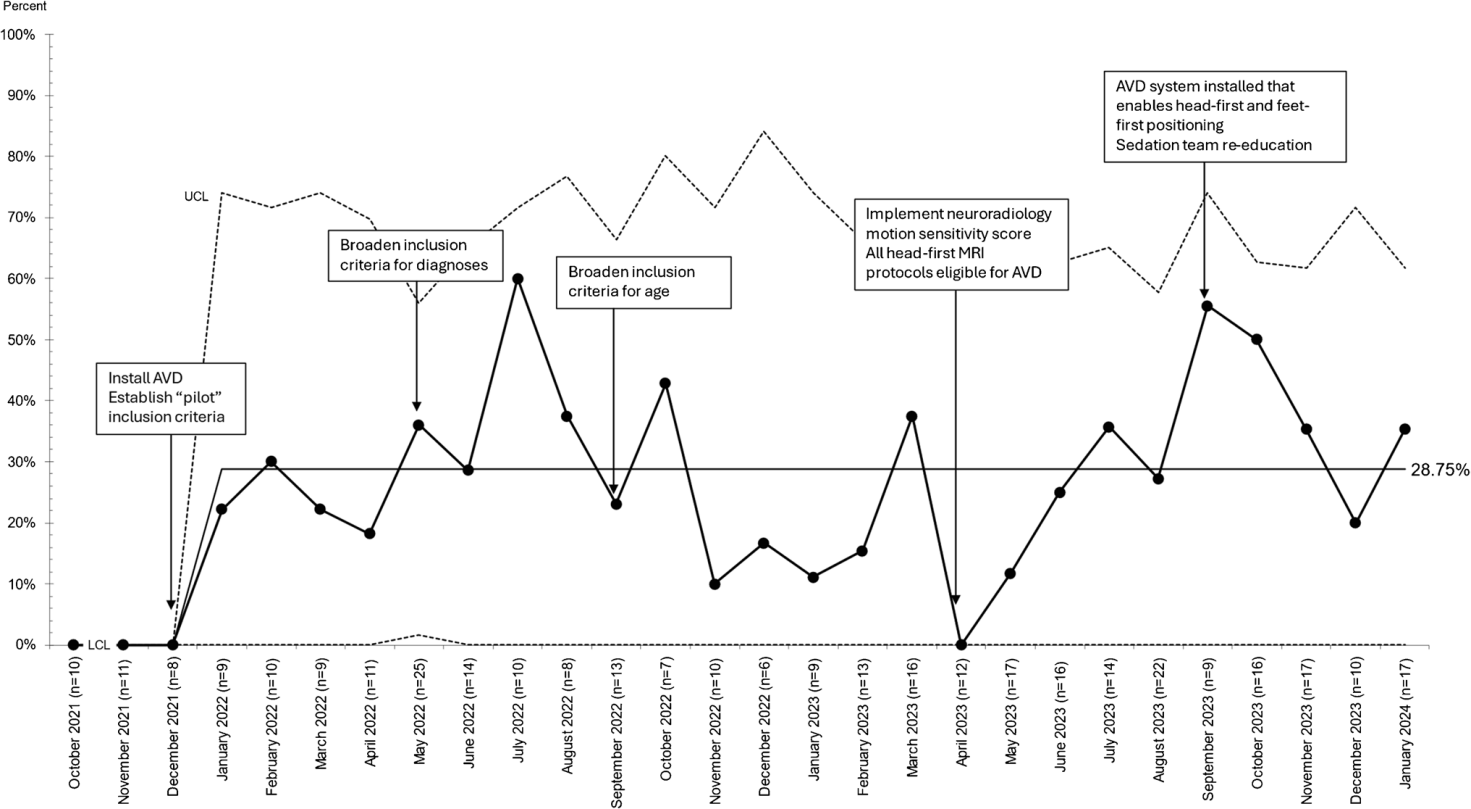
SPC P-Chart. Primary outcome measure: percentage of patients completing magnetic resonance imaging with audiovisual distraction and without sedation

**Fig. 3:**
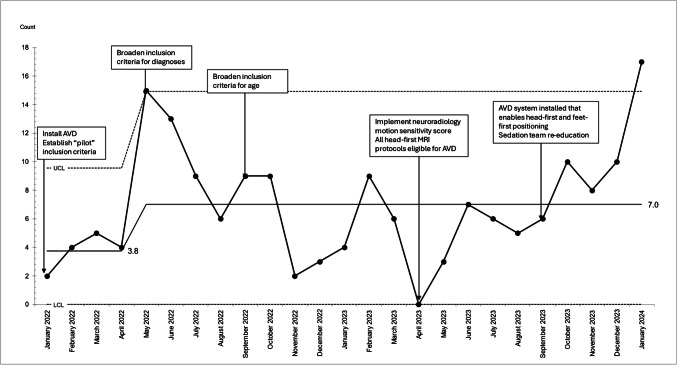
SPC C-Chart. Process measure: number of patients meeting eligibility criteria for audiovisual distraction

Of 1,015 patients scheduled for a sedated MRI through our clinic, 31.5% (*n* = 320) met study inclusion criteria of being 4 to 18 years of age, scheduled for MRI at the pediatric hospital, and eligible for either minimal/moderate sedation or AVD (Fig. 1). Of 320 patients meeting study inclusion criteria, 71.3% (*n* = 228) had minimal/moderate sedation and 28.8% (*n* = 92) had AVD without the need for sedatives. After 25 months of interventions following the introduction of AVD, the average monthly decrease in minimal and moderate sedation use was 28.8 (absolute) percentage points (Fig. 2). Over the study period, the average number of eligible patients per month increased by special cause variation from 3.8 to 7 patients per month (Fig. 3).

Table 4 shows the demographics and clinical characteristics of patients triaged to awake MRI with AVD (*n* = 92) compared to minimal or moderate sedation (*n* = 228). There was no statistical difference in age between patients triaged to AVD versus sedation, age years mean (SD) 10.0 (± 2.7) and 9.7 (± 3.0), respectively, *P* = 0.34. All 92 MRI referrals triaged to AVD completed their MRI successfully without sedation; all studies were of diagnostic quality, including 10 children with developmental delay and three with autism spectrum disorder. Most MRIs were completed within the allotted exam time in both the AVD group (95.7%; *n* = 88) and the sedation group (98.2%; *n* = 224), with no statistically significant difference between groups (*P* = 0.29).

**Table 4:** Demographics and clinical characteristics of awake and minimal and moderate sedated MRI referrals

Characteristic	Audiovisual distraction (*n* = 92)	Minimal/moderate sedation (*n* = 228)	*P*-value
Age (years)			0.34
Mean (SD)	10.0 (2.7)	9.7 (3.0)	
Median [IQR]	10 [8, 12]	9 [8, 12]	
Range	4–16	4–17	
Age group, *n* (%)			0.41
4–8 years	29 (31.5)	83 (36.4)	
9–18 years	63 (68.5)	145 (63.6)	
Sex, *n* (%)			0.32
Female	42 (45.7)	118 (51.8)	
Male	50 (54.3)	111 (48.2)	
Type of MRI, *n* (%)			**0.03**
Brain/neck/orbit/face	68 (73.9)	133 (58.3)	
Spine	12 (13.0)	55 (24.1)	
Extremity	7 (7.6)	14 (6.1)	
Other	1 (1.1)	1 (0.4)	
2 or more body parts	4 (4.3)	25 (11.0)	
Motion sensitivity score			**0.04**
Mean (SD)	2.1 (0.75)	2.3 (0.83)	
Median (IQR)	2 [2,2]	2 [2,3]	
MRI duration (minutes)			0.07
Range	20–139	4–151	
Median [IQR]	45 [36–56]	49 [38–66]	
MRI duration within allotted exam time, *n* (%)			0.29
No	4 (4.3)	4 (1.8)	
Yes	88 (95.7)	224 (98.2)	

Values in bold indicate statistical significance

**Fig. 4:**
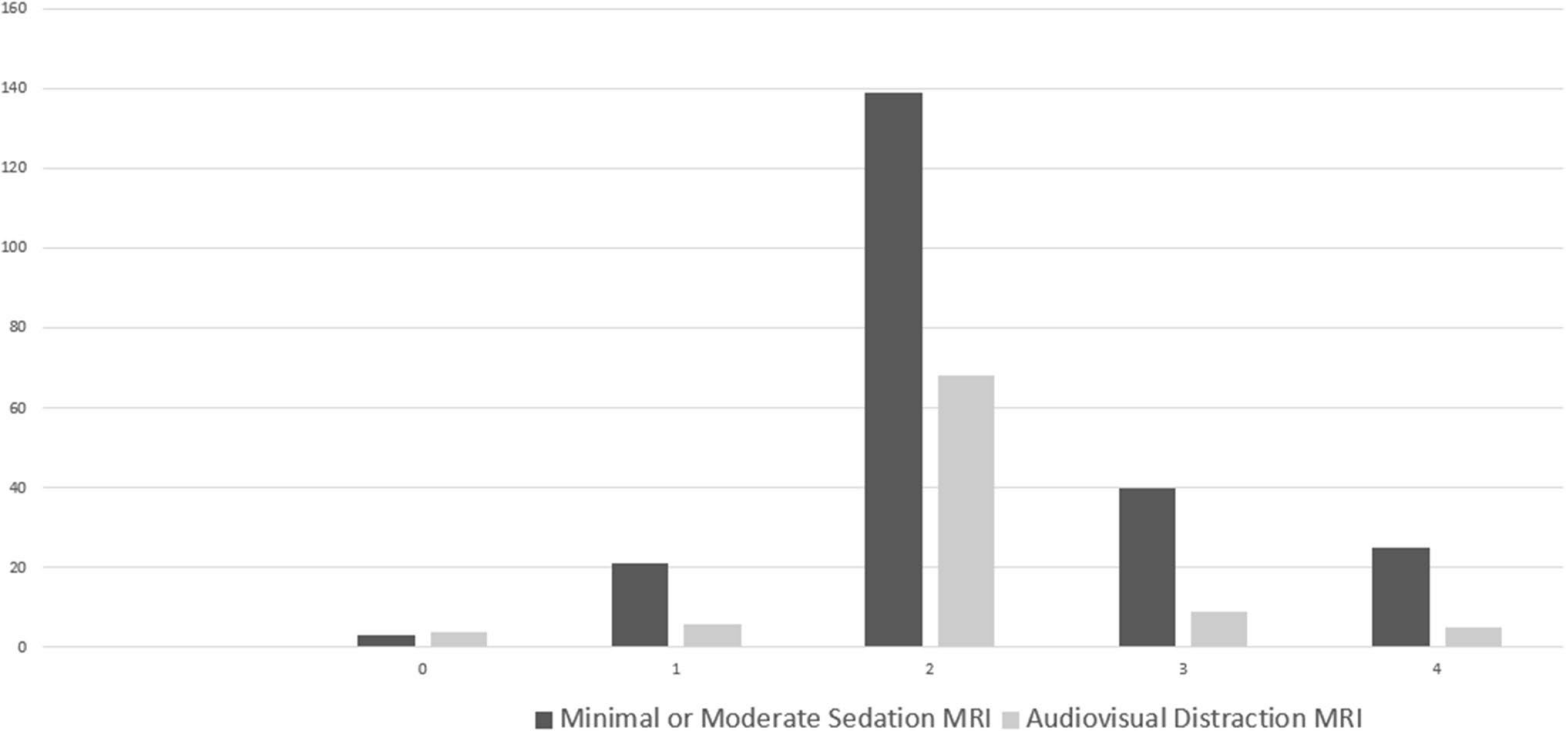
Bar chart illustrating relative distributions of motion sensitivity scores (0 through 4) between the minimal-moderate sedation and the AV distraction groups

The most common types of MRI in both the AVD and the minimal/moderate sedation group were brain/neck/orbit/face and spine. 86.7% (*n* = 80) of patients triaged to AVD and 82.5% (*n* = 188) of patients triaged to sedation had an MRI brain/neck/orbit/face or spine. The distribution of MRI exam types differed between those triaged to AVD or minimal/moderate sedation (*P* = 0.03), with a higher proportion of brain/neck/orbit/face in the AVD group (73.9%) compared to the sedation group (58.3%) and a higher proportion of spine (24.1% versus 13%) and two or more body parts (11% versus 4.3%) in the sedation group compared to the AVD group. Motion sensitivity scores were higher in patients triaged to minimal or moderate sedation, mean (SD) 2.3 (± 0.83) compared to 2.1 (± 0.75) for patients triaged to AVD, *P* = 0.04 (Fig. 4 and Table 5). There was no difference in MRI duration between AVD and sedated referrals, median (IQR) minutes, 45 (36–56) versus 49 (38–66), respectively (*P* = 0.07). There were no differences in other demographic and clinical characteristics between AVD and minimal or moderate sedated MRI referrals.

**Table 5:** Motion sensitivity score of MRI referrals triaged to audiovisual distraction or minimal/moderate sedation

Motion sensitivity score	Audiovisual distraction (*n* = 92)	Minimal/moderate sedation (*n* = 228)
0	4 (4.3)	3 (1.3)
1	6 (6.5)	21 (9.2)
2	68 (73.9)	139 (61)
3	9 (9.9)	40 (17.5)
4	5 (5.4)	25 (11)

**Fig. 5:**
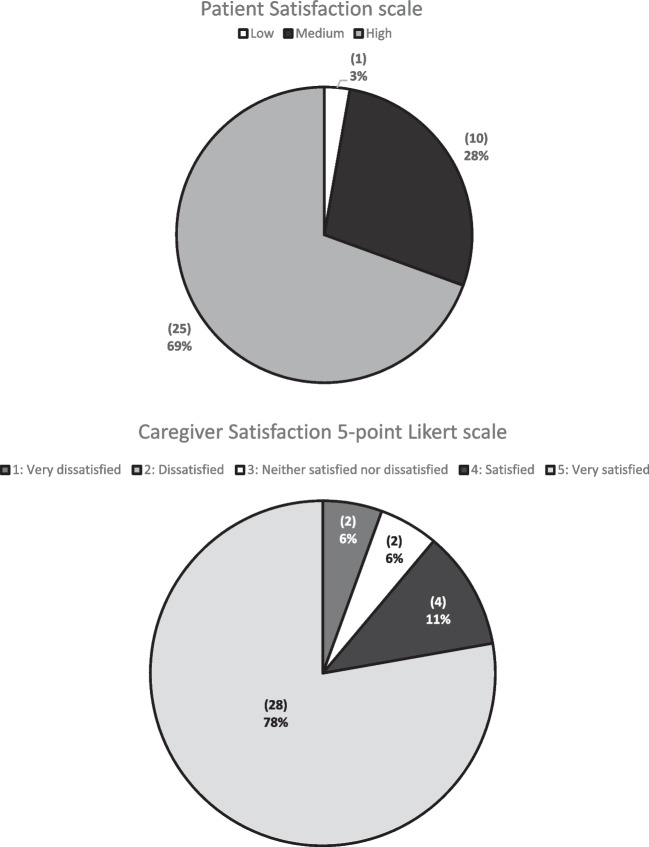
Patient and caregiver satisfaction survey results

A convenience sample of patients and caregivers completed 36 surveys rating the AVD MRI experience. Patients were highly satisfied and moderately satisfied in 69% and 28% of surveys, respectively. Caregivers were very satisfied and satisfied in 78% and 11% of the surveys, respectively (Fig. 5).

## Discussion

Using rigorous quality improvement methodology, we successfully implemented an awake MRI program using new open-bore AVD technology that decreased monthly average minimal and moderate sedation needs by 28.8 (absolute) percentage points, while maintaining a high rate of diagnostic studies (100%), while adhering to allotted exam times and maintaining high patient/parent satisfaction. Before implementing the awake MRI program with AVD, 698 MRI referrals would have required sedation. However, with the implementation of AVD technology, only 606 out of 698 MRI referrals required sedation. Therefore, AVD technology decreased the overall use of sedation during the study period by 13% (92/698).

Our study achieved special cause variation (i.e., a shift in the centerline) with the introduction of AVD technology in January 2022. The centerline (i.e., mean percentage of MRI referrals scanned awake with AVD) shifted from zero referrals before implementing AVD to 28.8% of MRI referrals, 4 to 18 years, after implementing AVD. This shift is depicted in Fig. 2, SPC P-Chart primary outcome measure, and identified by 8 consecutive points above the mean line. However, despite our initial conservative approach to awake MRI scanning and various PDSA cycles focusing on broadening the criteria for awake MRI scanning, we did not achieve a second shift in our centerline. These findings suggest that the introduction of AVD technology was the most important factor in reducing MRI sedation.

While brain/neck/orbit/face MRI exams were more often triaged to AVD, spine and two or more body part MRI exams were more often triaged to sedation. These differences are attributable primarily to technical factors: accelerated protocols are more common for various brain/neck/orbit/face MRI imaging, often associated with a lower difficulty or “motion sensitivity score” (Table 3), allowing for a higher predicted success rate with AVD; patient positioning within the scanning bore for spine and body imaging was not always compatible with the AVD system depending on patient size; and abdominal MRI exams that rely on breath-holds may steer a young patient to GA referral.

Previous studies reported a reduction in MRI sedation by 15–35% with the use of MRI goggles for distraction during MRI scanning. Harned et al. were the first to report a reduction in MRI sedation with the implementation of AVD technology [11]. The study compared the rate of MRI sedation before (49%) and after (40%) introducing MRI-compatible goggles. There was an 18% reduction in the percentage of patients requiring sedation after the introduction of AVD technology. However, the benefit was limited only to patients 3 years and older. There was a 17% reduction in MRI room time when non-sedated patients were compared to sedated patients. The study did not report the quality of awake AVD images, the AVD failure rate requiring sedation to complete a diagnostic MRI, or details on how patients were triaged to AVD versus sedation. A study by Khan et al. instituted a multicomponent “sedation reduction program” in children 7 years and younger, hiring a CCLS, introducing MRI goggles, and instituting a culture change emphasizing the need to avoid sedation for MRI [12]. There was a 34.6% decrease in the frequency of sedation for MRI, from 80.8% before the program to 52.8% after instituting the program. The sedation reduction was significant in children of all ages, including children 3 years and younger. However, the relative contribution of each component of the program to the observed reduction in MRI sedation was unclear. In addition, this study also did not report on the quality of awake AVD images, the AVD failure rate requiring sedation to complete a diagnostic MRI, or details on how patients were triaged to AVD versus sedation. A study by Lemaire et al. evaluated the impact of introducing MRI goggles on MRI wait times, image quality, and patient experience [13]. There was a 7.2% increase in pediatric patients scanned and a decrease of 15.4% in sedation utilization. The average sedation wait time decreased by 33% (5.8 months). Overall, the greatest impact was observed in the 4– to 10-year-old age group. Radiological evaluation revealed no difference in image quality between the AVD and sedated groups, and 84% of children expressed a positive reaction to the A/V system. However, the study did not report the AVD failure rate requiring sedation to complete a diagnostic MRI or details on how patients were triaged to AVD versus sedation. Finally, Rudder et al. evaluated the impact of an MRI sedation reduction program, including preparation by CCLS, use of a mock scanner, and MRI AVD, on MRI wait times for children between 4–12 years of age [17]. The study found a reduced MRI wait time for AVD MRI. The average (SD) number of days for an awake MRI was 15.4 (18.5) days, compared to 46.2 (15.7) days for the third-available appointment with sedation/anesthesia. Patients who had an MRI without sedation had good image quality, with only 3.8% in their awake MRI program requiring rescheduling with sedation/anesthesia because of motion artifact.

Our study may provide a more accurate estimate of the impact of AVD technology in decreasing MRI sedation in children. First, we used rigorous quality improvement methodology involving the sedation clinic and the Department of Radiology, which has not previously been used in the literature evaluating awake MRI programs. Second, we reported our experience over more than 2 years of use of AVD technology. Finally, our intervention targeted the population most likely to benefit from AVD technology, namely children 4 years and older, who can understand the use of AVD technology and usually require lighter levels of sedation to remain motionless during MRI.

Most studies assessing the impact of AVD on MRI sedation needs have not reported the impact on MRI workflow [9–11]. MRI scanning interruptions or repeated sequences, because of patient movement, may extend the study time beyond the allotted exam time and affect MRI workflow. Similarly, failure to accomplish an awake diagnostic MRI requires the patient to be sedated at the same appointment or rescheduled with sedation/anesthesia. Rudder et al. reported that 3.8% of patients 4 years and older, in their awake MRI program, required rescheduling with sedation/anesthesia because of motion artifact affecting diagnostic quality [17]. We used rigorous quality improvement methodology, reporting MRI duration and showing all awake studies were of diagnostic quality and most within the allotted exam time, similar to our sedated patients, without impacting MRI workflow.

Most published sedation reduction studies used MRI goggles, a fully immersive audiovisual technology, which may be more effective than “open-bore” technologies. However, we used two different open-bore AVD MRI technologies, not previously studied, because of cost and cleaning convenience. We found these new technologies to be cost-effective and highly rated by our patients/families.

Our study has several limitations. A major limitation was our “selective criteria” for awake MRI scanning based on primary concerns of diagnostic imaging quality and secondary concerns of negatively impacting MRI workflow. Children younger than 4 years, with mild developmental delay/disabilities, or mild anxiety may still benefit from an awake MRI program [10]. A more liberal approach could have resulted in fewer sedated patients. However, our conservative approach optimized imaging diagnostic quality, minimized MRI delays, rescheduling with sedation/anesthesia, and overall impact on MRI workflow. In addition, we implemented numerous changes in MRI protocols and increased allotted exam time during the study, which could have impacted the use of sedation for MRI. Our awake MRI workflow was labor-intensive, requiring screening and in-person assessment by a CCLS of every patient referred to our sedation clinic for MRI. This resource-intensive workflow may not be suitable for resource-limited settings. Patient and caregiver satisfaction were collected from a convenience sample and were asked verbally to the patient rather than providing an objective scale. In addition, the satisfaction survey lacked anonymity and patients and caregivers may feel compelled to answer positively. This introduces obvious bias in the survey results. We used two new open-bore AVD technologies that were more cost-effective than available MRI-compatible goggles, but not fully immersive, and may be less effective than MRI goggles. Finally, we did not study the impact of our awake MRI program on sedation clinic appointment wait times, MRI wait times, or the number of patients scanned.

## Conclusion

We successfully implemented an awake MRI program that decreased average monthly minimal and moderate sedation needs by 28.8 (absolute) percentage points and overall sedation needs by 13% during the study period, while maintaining a high rate of diagnostic studies, no significant MRI delays, and high family satisfaction.

## Data Availability

Data supporting this study will be provided by the corresponding author upon request.
